# Identification of Novel Cerebrospinal Fluid Biomarkers for Cognitive Decline in Aneurysmal Subarachnoid Hemorrhage: A Proteomic Approach

**DOI:** 10.3389/fncel.2022.861425

**Published:** 2022-05-06

**Authors:** Fan Liu, Yun Bao, Binghui Qiu, Jian Mao, Xixian Liao, Haorun Huang, An Zhang, Guozhong Zhang, Songtao Qi, Fen Mei

**Affiliations:** Department of Neurosurgery, Nanfang Hospital of Southern Medical University, Guangzhou, China

**Keywords:** biomarker, cerebrospinal fluid, cognitive impairment, proteomics, subarachnoid hemorrhage

## Abstract

**Background:**

Cognitive impairment commonly occurs in aneurysmal subarachnoid hemorrhage (aSAH) survivors. Cerebrospinal fluid (CSF) biomarkers have been proven useful in several central neurological disorders. No such diagnostic biomarkers are available for predicting cognitive impairment after aSAH to date. Here, we aimed to identify novel CSF biomarkers for cognitive deficits after aSAH using an in-depth proteomic approach.

**Methods:**

We applied mass spectrometry with data independent acquisition (DIA) quantification to identify biomarker candidates in CSF samples from a well-characterized cohort comprising patients with impaired cognition (*n* = 9) and patients with intact cognition (*n* = 9). The potential biological processes and signaling pathways associated with differential proteins were analyzed using R software. The candidates were further validated in a larger independent cohort (*n* = 40) using ELISA. The diagnostic utility of these proteins was investigated by using receiver operating characteristic curve analysis.

**Results:**

In total, we identified 628 proteins. The discovery cohort revealed that 115 proteins were differentially expressed in cognitive impairment patients compared to patients with intact cognition (*P* < 0.05). Independent cohort replication confirmed NCAM2, NPTXR, NRXN2, RELN, and CNTN2 as sensitive and specific candidate biomarkers for disorders of cognition. Lower CSF levels of all biomarker candidates, except RELN, were associated with more pronounced cognitive decline.

**Conclusion:**

We identified and validated five CSF biomarkers for cognitive impairment in aSAH patients. These particular proteins have important predictive and discriminative potential for cognitive impairment in aSAH and could be potential targets for early disease intervention.

## Introduction

Aneurysmal subarachnoid hemorrhage (aSAH) is a life-threatening disease resulting from the rupture of intracranial aneurysms. aSAH accounts for only 5%–10% of strokes annually, but it disproportionately contributes to potential life lost due to long-term disability and mortality (Johnston et al., 1998; Rinkel and Algra, 2011). Many patients who have experienced aSAH increasingly survive the initial hemorrhage due to improvements in aSAH treatment and ICU care. However, aSAH has a significant impact on the ability to return to work and years of potential life lost (James et al., 2018).

One critical cause of long-term disability is cognitive impairment, and recent studies have shown that at least 50% of all aSAH patients who return to the community exhibit cognitive dysfunction (Mavaddat et al., 1999; Al-Khindi et al., 2010). Verbal memory, executive function, and language are the most commonly impaired cognitive domains (Kreiter et al., 2002; Wong et al., 2012). Various mechanisms likely influence the cognitive function of patients who have experienced aSAH, including cerebral vasospasm, microvascular dysfunction, neuroinflammation, microthrombosis, and electrophysiologic abnormalities (Kranc et al., 2000; Stein et al., 2006; Vergouwen et al., 2008; Sabri et al., 2012; Geraghty and Testai, 2017). Collectively, however, the current understanding of the pathophysiology of cognitive impairment in aSAH patients is still incomplete. Thus, it is imperative to identify and validate new biomarkers for cognitive impairment that could provide a convenient, quantifiable, and biological measure of disease progression.

Cerebrospinal fluid (CSF) is the best matrix to identify novel biomarkers for central nervous system disorders due to its direct contact with the brain parenchyma and biochemical alterations in the brain that are reflected in CSF (Al Shweiki et al., 2017; Norwood et al., 2019). CSF biomarkers have been proven useful in several disorders, such as depressive disorder and Alzheimer’s disease (AD; Bereczki et al., 2018; Al Shweiki et al., 2020). The development of CSF biomarkers to assist in early differential diagnosis and predicting disease progression from its earliest stage is of major importance for both research and therapeutic development. To date, no such diagnostic biomarkers are available for aSAH-induced cognitive impairment. Compared to genetics and transcriptomics, proteomics measures various proteins that represent the major functional molecules in a biological system, and hence, proteomic profiling provides a closer approximation of the dynamic pathophysiological processes.

In this study, we investigated the CSF proteome of aSAH patients, focusing on cognitive dysfunction, by using a deep proteomic profiling approach combined with a further validation step using enzyme-linked immunosorbent assays (ELISAs). We hypothesized that these candidate proteins in CSF may be involved in the cognitive decline process in aSAH patients.

## Materials and Methods

### Patients and CSF Collection

A single-center, prospective study was conducted on patients with acute nontraumatic SAH in the Department of Neurosurgery at Nanfang Hospital of Southern Medical University in Guangzhou, China. Adult patients (aged 20–60 years) of both sexes, hospital admission within 24 h of ictus, presented with spontaneous aneurysmal SAH, diagnosed by initial brain computed tomography (CT) at onset, and confirmed by digital subtraction angiography and/or computerized tomography angiography within 1–3 days of onset were included in the study. All patients treated by endovascular coiling by a single cerebrovascular neurosurgeon. Patients were excluded from this study in the following cases: a history of previous cerebrovascular or neurological disease other than unruptured intracranial aneurysm, marked impaired consciousness, previous dementia, marked hepatic or renal impairment, or patients with nonaneurysmal SAH and disability in cognitive assessment, such as severe aphasia, deafness or difficulty in completing the Montreal Cognitive Assessment (MoCA). None of patients developed any further neurological complications other than aSAH such as cerebral hemorrhage or stroke. This study was approved by the Nanfang Hospital of Southern Medical University Institutional Review Board.

For the biomarker discovery phase, the CSF of 20 patients was selected from September 2019 to January 2020 according to the criteria described above and used for mass spectrometry. Because two samples failed in library construction, the actual execution was 18 samples, including nine intact cognition samples and nine impaired cognition samples, in proteomic profiling. For the validation phase, a second cohort consisting of an independent set of 48 aSAH patients was selected from August 2020 to April 2021. During the 6-month follow-up period, contact was lost in eight cases. The final cohort included 40 patients, containing 21 intact cognition samples and 19 impaired cognition samples, to validate the identified candidate biomarkers using ELISA. All CSF samples were obtained by the first time of lumbar puncture within 48 h of onset and placed in a 15-ml RNase/DNase-free centrifuge tube. The whole CSF was centrifuged at 5,000 *g* at 4°C for 10 min and the supernatant was taken, transferred to polypropylene tubes of 0.5 ml and stored at −80°C until further analysis.

### Baseline Clinical Characteristics

Demographic and clinical data on age, sex, current smoking, and drinking status, the location of aneurysms, systolic and diastolic blood pressures and laboratory tests of CSF were collected at study onset. A previous history of diabetes was recorded *via* self-reports. Additionally, a complete neurological examination was carried out for all cases, including the initial Glasgow Coma Scale (GCS) score and Hunt and Hess scale grades, and the severity of aSAH was detected by using the modified Fisher scale. Cerebral vasospasm was determined *via* transcranial Doppler ultrasound findings or cerebral angiography using previously described criteria or diagnosis and evaluation by the neurosurgeon (Marshall et al., 2010).

### Cognitive Assessment at Follow-Up

During the follow-up of patients, cognitive function was evaluated by using the Chinese version of the MoCA 6 months after the onset of SAH (Nasreddine et al., 2005; Bateman et al., 2016). The scores range from 0 to 30, with higher scores representing better cognitive function. The diagnosis of cognitive impairment was defined as MoCA scores less than 26. According to MoCA scores, patients were classified into two principal groups: cases with cognitive impairment (MoCA scores <26) and cases with intact cognitive function (MoCA scores ≥26).

### Sample Preparation and Gel Electrophoresis

CSF samples were coded and analyzed in a blinded fashion. An SDT lysis buffer was added to the sample. The lysate was sonicated and then boiled for 15 min. After centrifugation at 14,000 *g* for 40 min, the supernatant was quantified with a BCA Protein Assay Kit (P0012, Beyotime). The sample was stored at −20°C. Twenty micrograms of protein calculated from each sample were mixed with 6× loading buffer and boiled for 5 min. The proteins were separated on a 12.5% SDS–PAGE gel. Protein bands were visualized with Coomassie Blue R-250 staining.

### NanoLC–MS/MS (Nanoscale Liquid Chromatography Coupled to Tandem Mass Spectrometry) Analysis

The separated proteins were subjected to in-gel digestion before nanoLC–MS/MS analysis. The peptides were redissolved in solvent A (A: 0.1% formic acid in water) and analyzed by online nanospray LC–MS/MS on an Orbitrap Exploris 480 coupled to an EASY-nLC 1200 system (Thermo Fisher Scientific, MA, USA). Two microliters of peptide sample were loaded on an analytical column (Thermo Fisher Scientific, Acclaim Pep Map RSLC 50 μm × 15 cm, nanoViper, P/N164943) and separated using B (B: 0.1% formic acid, 80% ACN), gradient from 5% to 38% for 120 min. The column flow rate was maintained at 300 nl/min. An electrospray voltage of 2 kV relative to the inlet of the mass spectrometer was used. The mass spectrometer was run in data-independent acquisition mode and automatically switched between MS and MS/MS modes. The parameters were as follows: (1) MS: scan range (m/z) = 350–1,500; resolution = 60,000; automated gain control (AGC) target = 3e6; maximum injection time = 50 ms; (2) Higher-energy C-trap dissociation (HCD)–MS/MS: resolution = 30,000; AGC target = 1e6; collision energy = 28; and (3) Data-independent acquisition (DIA) was performed with a variable isolation window, each window overlapped 1 m/z, the window number was 42, and the total cycle time was 3 s.

### Protein Identification and Quantification

Raw DIA data were processed and analyzed by Spectronaut X (Biognosys AG, Switzerland) with default settings, and the retention time prediction type was set to dynamic indexed retention time (iRT). Data extraction was determined by Spectronaut X based on extensive mass calibration. Spectronaut Pulsar X will dynamically determine the ideal extraction window depending on iRT calibration and gradient stability. A Q value (false discovery rate, or FDR) cutoff at the precursor and protein levels was set to be 1%. Decoy generation was set to be mutated, which was similar to “scrambled” but merely applied with a random number of AA position swaps (min = 2, max = length/2). All selected precursors passing the filters were used for quantification. MS2 interference will remove all interfering fragment ions except for the three least interfering ions. The average top three filtered peptides that passed the 1% Q value cutoff were used to calculate the major group quantities. Protein abundance was quantified by the area under the chromatographic peak of MS peptide precursor ion signal intensity. Student’s *t*-test was performed, and proteins were defined as differentially expressed if they had a *p* value < 0.05 and a fold change >1.5.

### ELISAs

ELISA kits for each of the selected proteins were procured from Cloud-Clone Corporation (USA), and experiments were carried out according to the manufacturer’s instructions. Standard samples (100 ml) for catalase (CAT), vimentin (VIM), amyloid beta precursor protein (APP), neural cell adhesion molecule 2 (NCAM2), amyloid beta precursor like protein 1 (APLP1), reelin (RELN), contactin 2 (CNTN2), and neurexin (NRXN2) were incubated with the corresponding HRP-conjugated reagent for 1 h at 37°C, followed by thorough washing steps. A chromogen solution was then applied, and immediately after the reaction was stopped, absorbance was measured at 450 nm on a microplate reader within 15 min. The amount of sample was obtained using the fitted standard curve, and standards/samples were measured in duplicate.

### Receiver Operating Characteristic (ROC) Curve Analysis

**Receiver operating characteristic (ROC)** curves were constructed to assess the predictive value of the candidate protein by calculating the area under the curve (AUC) values in aSAH patients. The sensitivity and specificity of the prognostic characteristic of candidate proteins in predicting clinical outcome were evaluated by calculating the AUC value of the ROC curve using Prism 8.0 software.

### Statistical Analysis

Categorical variables were expressed as percentages of the group, and continuous variables were expressed as the median and interquartile range or mean and standard deviation (SD) depending on their distribution. The chi-square test and Fisher’s exact test were used to assess categorical variables. Continuous variables with normal distributions were compared using independent Student’s *t*-test, and those not normally distributed were evaluated by the nonparametric test. A *p* value < 0.05 was deemed statistically significant. Correlations between the expression level of candidate proteins in CSF and MoCA scores were assessed with the Spearman partial correlation coefficient. All statistical tests were performed using SPSS for Windows, version 22.0 (SPSS, Chicago, IL, USA).

## Results

### General Characteristics of the Study Population

The baseline characteristics of the participants with and without cognitive impairment included in the development cohort and validation cohort are presented in Table 1. The two cohorts had similar age and sex distributions. The MoCA scores were <26 in 48.3% (28/58) of patients defined as having cognitive impairment. In the validation cohort, cases with impaired cognition in comparison with cases with intact cognitive functions showed significantly higher modified Fisher scale scores (*p* = 0.004), while diabetes, smoking, drinking, blood pressure, GCS, Hunt-Hess grade, location of aneurysm, the incidence of cerebral vasospasm, and laboratory tests of CSF were not significantly different between the two groups. The total MoCA scores of aSAH patients with cognitive impairment were significantly lower than those of patients with intact cognition (22.86 ± 1.9 vs. 27.07 ± 0.94, *p* < 0.001). Additionally, there was a significant impairment in the subtests of the MoCA, especially the domains of visuospatial/executive ability, attention, language, and delayed recall, while the domains of naming, abstraction, and orientation were not significantly diminished after impaired cognition in aSAH patients (Table 2).

**Table 1:** Baseline characteristics of the patients with aneurysmal subarachnoid hemorrhage.

	**Development cohort (*n* = 18)**			**Validation cohort (*n* = 40)**		
	**Impaired cognition (*n* = 9)**	**Intact cognition (*n* = 9)**	***p* value**	**Impaired cognition (*n* = 19)**	**Intact cognition (*n* = 21)**	***p* value**
Age, years	49.9 ± 8.8	48.8 ± 9.9	0.804	50.6 ± 7.2	49.9 ± 7.8	0.793
Gender			1			0.488
Male	2 (22.2)	3 (33.3)		4 (21.1)	7 (33.3)	
Female	7 (77.8)	6 (66.7)		15 (78.9)	14 (66.7)	
Diabetes, %	3 (33.3)	0 (0)	0.206	2 (10.5)	3 (14.3)	1
Smoking, %	1 (11.1)	3 (33.3)	0.576	2 (10.5)	5 (23.8)	0.412
Drinking, %	1 (11.1)	0 (0.0)	1	1 (5.3)	5 (23.8)	0.186
Hypertension, %	6 (66.7)	4 (44.4)	0.637	13 (68.4)	12 (57.1)	0.527
GCS, score	14 (13.5–15)	15 (13.5–15)	0.737	14 (11–15)	14 (13.5–15)	0.766
Modified Fisher score	3 (2–3.5)	3 (1.5–3)	0.739	3 (3–4)	2 (2–3)	**0.004**
Hunt-Hess grade	2 (1–2.5)	2 (1.5–3)	0.539	2 (2–3)	2 (2–3)	0.135
Aneurysm location, %			0.14			0.359
ACA/ACom	5 (50)	5 (50)		7 (36.8)	8 (38.1)	
ICA/PCom	4 (44.1)	1 (11.1)		5 (26.3)	8 (38.1)	
MCA	0 (0)	3 (33.3)		4 (21.1)	5 (23.8)	
Posterior	0 (0)	0 (0)		3 (15.8)	0 (0)	
Cerebral vasospasm, %	6 (66.7)	4 (44.4)	0.637	13 (68.4)	13 (50)	0.748
CSF examination						
WBC, *n*/μl	130.5 ± 119.5	117.7 ± 95.3	0.716	118.5 ± 100.8	94.3 ± 107.1	0.469
Monocytes, *n*/μl	56.9 ± 23	48.5 ± 26.6	0.486	37.9 ± 25.8	40.6 ± 30.5	0.764
RBC, *n* × 10^4^/μl	9.4 ± 10.8	6 ± 4.1	0.397	5.5 ± 5.3	6.9 ± 6.8	0.472
Protein concentration, g/L	0.8 ± 0.5	0.6 ± 0.2	0.317	0.7 ± 0.4	0.6 ± 0.4	0.485
Length of stay, days	14 (13–18.5)	14 (13–17)	0.964	16 (14–20)	14 (12–17.5)	0.103

*Data are presented as the mean ± SD for normally distributed continuous variables, as the median [first quartile − third quartile] for nonnormally distributed continuous variables or as *n* (%) for categorical variables. GCS, Glasgow Coma Scale; ACA, anterior cerebral artery; ACom, anterior communicating artery; ICA, internal carotid artery; PCom, posterior communicating artery; MCA, middle cerebral artery; Posterior, posterior circulation including vertebral and basilar arteries and their branches; WBC, White blood cell; RBC, Red blood cell. The bold value indicates a significant difference was observed between groups*.

**Table 2:** Classification of patients with aSAH based on cognitive impairment.

	**Impaired cognitive function**	**Intact cognitive function**	***p* value**
Number	28 (48.3)	30 (51.7)
MoCA scores			
Visuospatial/executive ability	3.89 ± 0.88	4.5 ± 0.68	**0.005**
Naming	2.61 ± 0.5	2.8 ± 0.41	0.111
Attention	3.86 ± 0.97	5.07 ± 0.74	**<0.001**
Language	2.21 ± 0.42	2.8 ± 0.41	**<0.001**
Abstraction	1.71 ± 0.46	1.9 ± 0.31	0.074
Delayed recall	3.57 ± 0.57	4.73 ± 0.52	**<0.001**
Orientation	5 ± 0.72	5.23 ± 0.43	0.137
Total score	22.86 ± 1.9	27.07 ± 0.94	**<0.001**

*aSAH, aneurysmal subarachnoid hemorrhage; MoCA, montreal cognitive assessment. The bold value indicates a significant difference was observed between groups*.

### Discovery Proteomic Analysis of CSF Obtained From aSAH Patients Focusing on Cognitive Function

**Figure 1:**
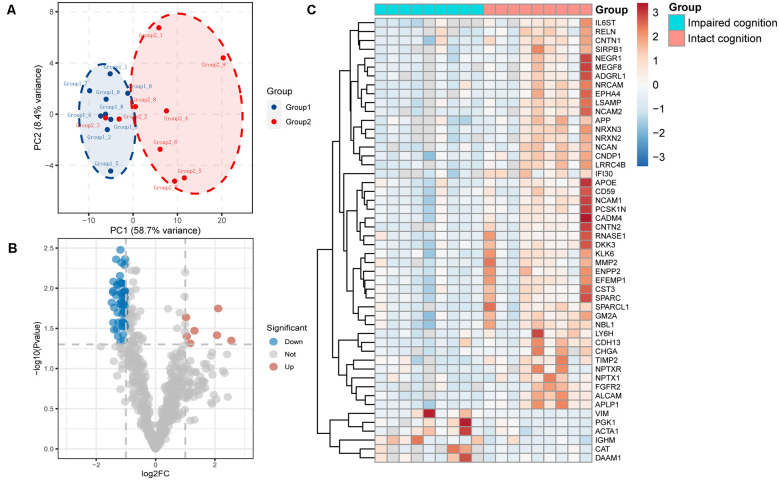
Proteomic data analysis within the impaired cognition and intact cognition groups. **(A)** PCA of the two groups. The PCA plot shows principal components 1 and 2 (PC1 and PC2), which explain 58.7% and 8.4% of the total variance, respectively. **(B)** Volcano plot shows differentially expressed proteins between the two groups. The horizontal axis indicates log_2_(fold change). The vertical axis indicates −10 log *p* values. Each dots represents a protein, dots in red and blue have a fold change >1.5 and *p* value < 0.05 analyzed by Student’s *t*-test. The gray dots represent proteins with no statistically significant difference in expression. **(C)** Heatmap of mass spectrometry data from CSF samples of individuals in the impaired cognition group (*n* = 9) and the intact cognition group (*n* = 9). Red indicates upregulation, and blue indicates downregulation.

We used principal component analysis (PCA) to detect the differences between the proteins contained in each array dataset. Simplifying this highly multidimensional data set into two-dimensional components enables unbiased comparison and visualization of the general condition of proteins between samples. The results showed that the impaired cognition group and intact cognition group could be differentiated significantly at the protein expression level. This result demonstrated that impaired cognition in aSAH patients may be closely related to proteomics in CSF, and the protein in CSF may represent a potential biomarker for predicting impaired cognition (Figure 1A).

To screen candidate biomarkers in CSF, we analyzed the differences in proteins in the CSF between the intact cognition group and the impaired cognition group through proteomics. The deep discovery proteomic analysis of 18 samples identified 628 protein groups. A total of 115 proteins were differentially regulated in the CSF between the two groups, specifically, 14 upregulated proteins and 101 downregulated proteins with the threshold of *p* values < 0.05 and |fold change| >1.5. The significance level and the magnitude of changes in the quantitative data are visualized in a volcano plot (Figure 1B). Forty-one downregulated and six upregulated known proteins in the impaired cognition group were detected in more than 80% of the samples in at least one group and are displayed as a heatmap to show the expression status of proteins in each sample between the two groups (Figure 1C).

**Figure 2:**
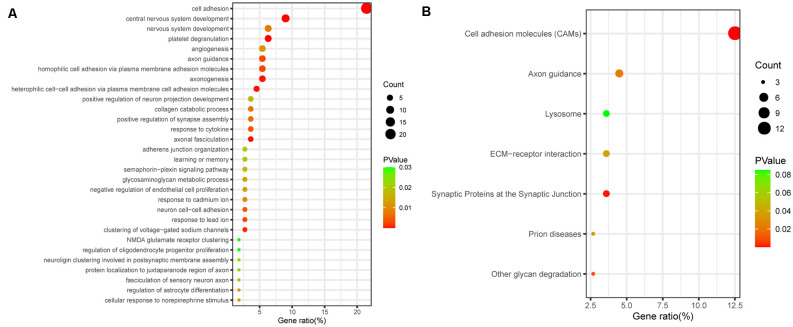
Bioinformatic analysis of all differentially expressed proteins. **(A)** GO analysis of proteomic data. The bubbles represent the top 30 biological processes related to cognitive dysfunction, *p* < 0.05. **(B)** KEGG analysis of proteomic data. The bubbles represent the seven enriched pathways, indicating their enrichment scores, gene counts, and *p* values.

Next, to further elucidate the potential functions of differentially regulated proteins, we investigated the potential biological processes and pathway analyses by the Kyoto Encyclopedia of Genes and Genomes (KEGG) database and Gene Ontology (GO) database. Using the Database for Annotation, Visualization and Integrated Discovery (DAVID^1^), we performed GO functional enrichment analysis and identified 141 significant GO terms, including 79 biological process (BP) terms, 40 CC terms, and 21 molecular function (MF) terms. The top 30 biological process terms are shown in Figure 2A, including cell adhesion, central nervous system development, axon guidance, axonogenesis, learning and memory, and NMDA glutamate receptor clustering (Figure 2A). According to the KEGG enrichment analysis, the enrichment pathway results showed that cell adhesion molecules, axon guidance, and synaptic proteins at the synaptic junction, which are involved in cognitive function, were significantly enriched (Figure 2B). Functional enrichment analysis of target genes further demonstrated that cognitive dysfunction in aSAH patients may be closely related to differential proteins in CSF.

### Validation of Candidate CSF Biomarkers

**Figure 3:**
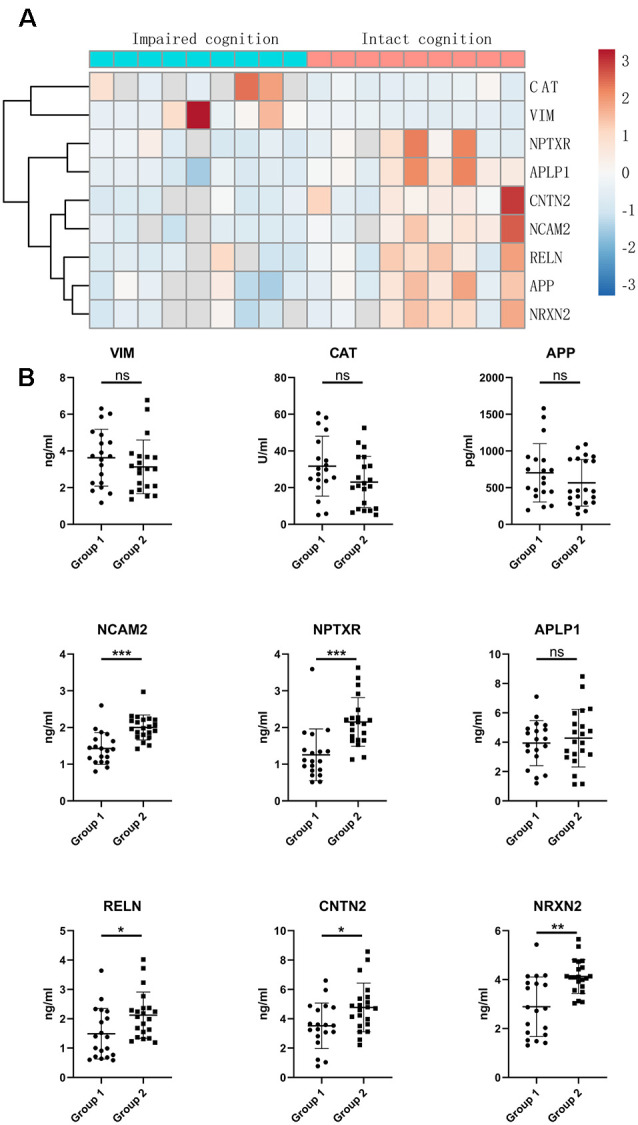
Validation of candidate proteins in the CSF of aSAH patients by ELISA. **(A)** Heatmap of candidate proteins that significantly differed between the two groups. **(B)** CSF levels of the candidate miRNAs in the impaired cognition group (*n* = 19) compared to the intact cognition group (*n* = 21). Group 1: impaired cognition; Group 2: intact cognition; ns, not significant; asterisks refer to statistically significant differences with *T* test, *p < 0.05; **p < 0.01; ***p < 0.001.

To validate the proteomic changes identified in the discovery experiment, we specifically measured several proteins in a larger sample cohort. The verified cohort consisted of 19 patients with impaired cognitive functions and 21 patients with intact cognitive functions. Target proteins that are significantly correlated with cognitive function were selected as candidate biomarkers and subsequently subjected to quantitative validation. We selected nine proteins (APP, CAT, NCAM2, NPTXR, APLP1, RELN, CNTN2, VIM, and NRXN2) for further verification by ELISA. We selected these specific proteins from the proteins that showed differential expression in the proteomics based on a literature analysis focusing on the following points: the magnitude (in terms of fold change) and significance of the difference in expression in CSF, protein expression enrichment in the brain, protein function, antibody availability, and/or a previous description of a possible association with cognitive function. The heatmap indicates that the expression of target proteins is different in the intact cognition group and the impaired cognition group (Figure 3A).

**Figure 4:**
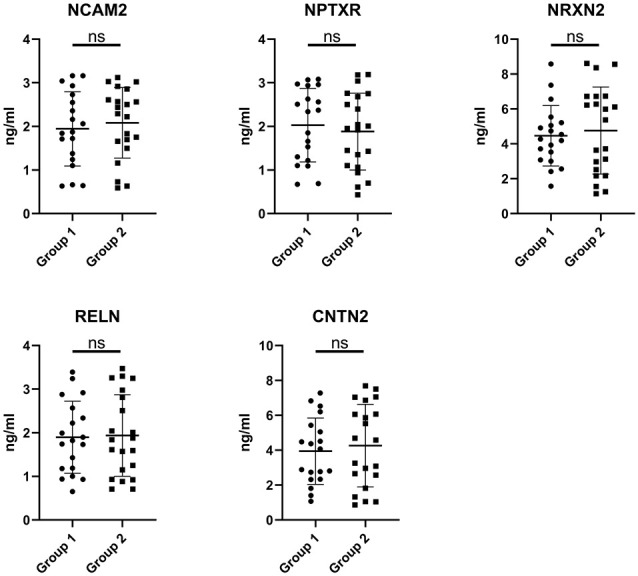
Blood levels of the candidate proteins in the impaired cognition group (*n* = 19) compared to the intact cognition group (*n* = 21). Group 1: impaired cognition; Group 2: intact cognition; ns, not significantly analyzed by Student’s *t*-test.

**Figure 5:**
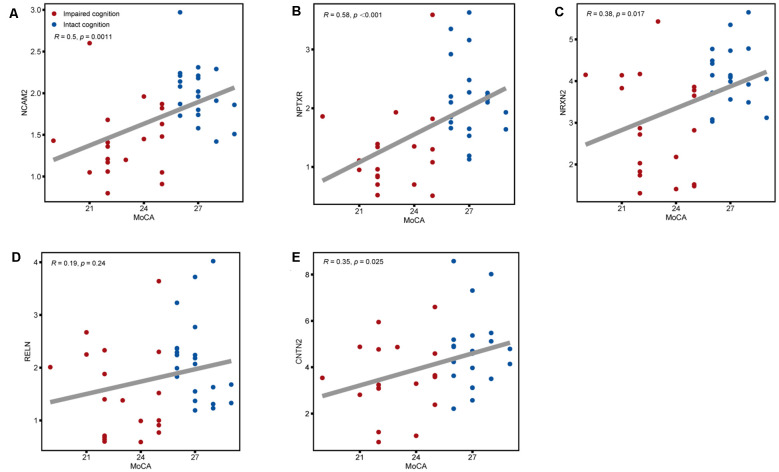
Associations between the five CSF candidate biomarkers for impaired cognition and actual scores on the MoCA. Scatter plots of MoCA scores and CSF levels of **(A)** NCAM2, **(B)** NPTXR, **(C)** NRXN2, **(D)** RELN, and **(E)** CNTN2 across the impaired cognition group (red) and the control group (blue). Associations were assessed using Spearman partial correlations adjusted for cohort. To correct for multiple comparisons, *p* values were corrected using a false discovery rate (FDR) correction.

The expression of the nine candidate proteins from the screening phase was further analyzed in the CSF of a larger cohort of aSAH patients using ELISA. As shown in Figure 3B, five proteins (NCAM2, NPTXR, NRXN2, RELN, and CNTN2) were still downregulated in the impaired cognition group (*p* < 0.05). To exclude the potential bias from contamination with peripheral circulation, the expression of these five proteins in the blood was also measured. We did not find any significant differences in their expression levels in the blood between the two groups (Figure 4). This finding indicates that the difference in the proteins identified originated in the central nervous system. In addition, the ELISA results revealed no significant difference in CAT, VIM, APP, and APLP1 levels. Next, we explored whether these five candidate biomarkers were associated with sex, age, and cognitive impairment. Partial Spearman correlation analysis adjusted for cohort revealed that lower CSF levels of all candidates except RELN were associated with lower MoCA scores at 6 months after the onset of SAH (0.37 < *r* < 0.56, all *p* < 0.05; Figure 5), whereas no association were found with sex and age (date not shown). In general, these differentially expressed proteins may be involved in cognitive dysfunction in aSAH patients.

### Diagnostic Value of the Identified Biosignature in CSF

**Figure 6:**
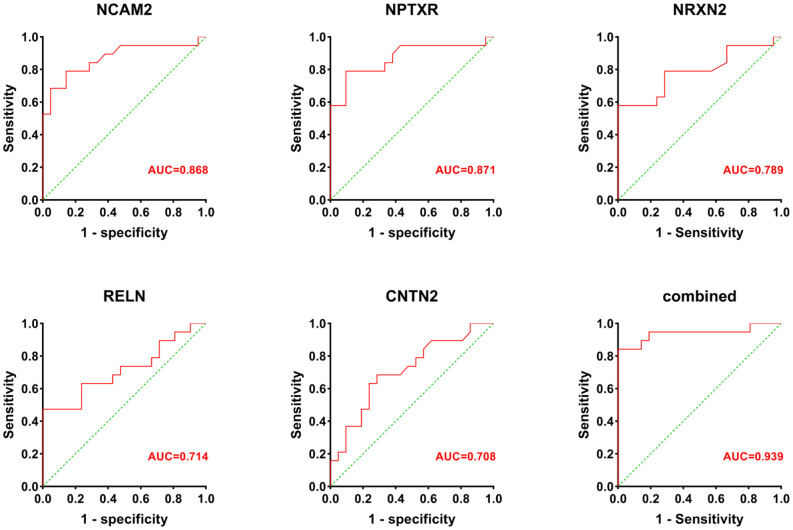
The AUC values (red numbers) of candidate biomarkers in ROC curve analysis. The combination of the five proteins had a stronger predictive value than any of the individual proteins.

Receiver operatin characteristic curve analysis was carried out to evaluate the predictive value of these five candidate biomarkers for cognitive dysfunction. First, we performed ROC analysis on a single protein. As shown in Figure 6, the ROC curve demonstrated that the AUC was 0.868 for NCAM2, 0.871 for NPTXR, 0.789 for NRXN2, 0.714 for RELN, and 0.708 for CNTN2 with a cutoff value, 95% confidence interval (CI), and sensitivity and specificity values, as shown in Table 3. Next, we combined five investigated candidates and constructed a panel for diagnosing cognitive impairment. The ROC curve demonstrated that the AUC was 0.939 (95% CI: 0.624–0.945; sensitivity = 84%, specificity = 100%; Figure 6). The combination of the five proteins had better performance than any of the five proteins alone, which indicated the better diagnostic ability of this panel compared with a single protein.

**Table 3:** Sensitivity, specificity, cutoff value, and AUC for each candidate biomarker.

	**Cutoff**	**AUC**	**Sensitivity**	**Specificity**	**95% CI**	***p* value**
CAT	>23.3	0.654	79%	57%	0.567–0.915	0.1
VIM	>3.69	0.615	53%	76%	0.549–0.894	0.213
APP	>383	0.589	85%	43%	0.624–0.945	0.336
NCAM2	<1.705	0.87	80%	86%	0.654–0.95	<0.001
NPTXR	<1.46	0.87	79%	90%	0.567–0.915	<0.001
APLP1	<5.85	0.524	95%	24%	0.754–0.997	0.797
RELN	<1.095	0.714	47%	100%	0.273–0.683	0.021
CNTN2	<3.81	0.708	68%	71%	0.46–0.846	0.025
NRXN2	<2.95	0.79	58%	100%	0.363–0.769	0.002

*AUC, area under the curve*.

## Discussion

In this study, we analyzed whether patients with aSAH developed cognitive dysfunction, and we divided the patients into two groups, an intact cognition group and an impaired cognition group, according to MoCA follow-up results at 6 months after surgery. Next, using a rigorous, state-of-the-art proteomic approach, we filtered nine promising CSF biomarker candidates for cognitive dysfunction. In a second validation phase, we confirmed by ELISA that five of the nine biomarker candidates, namely, NCAM2, NPTXR, NRXN2, RELN, and CNTN2, were also downregulated in a larger independent cohort of patients with impaired cognition compared to patients with intact cognition. Furthermore, we validated that these five proteins may serve as novel potential biomarkers for the diagnosis of cognitive disorder in aSAH patients with acceptable specificity and sensitivity. Moreover, our study revealed that lower CSF levels of all biomarker candidates, except RELN, were associated with lower MoCA scores at 6 months after the onset of SAH. To our knowledge, this is the most extensive analysis on the CSF proteome focusing on cognitive dysfunction in aSAH patients to date.

The application of key synaptic proteins as potential biomarkers has recently become the spotlight of discussions in various dementias (Bereczki et al., 2016, 2018; Wellington et al., 2016). There is increasing evidence suggesting synaptic dysfunction and impaired connectivity of certain brain circuits as molecular mechanisms underlying cognitive impairment in aSAH patients (Shen et al., 2015; van Dijk et al., 2016; Zhou et al., 2019). Consistently, animal studies on rodents revealed reduced synaptic density upon stress (Csabai et al., 2018). In this study, GO and KEGG term analyses associated the differentially regulated proteins in cognitive dysfunction patients with synaptic function. Furthermore, our discovery reveal significantly decreased CSF levels of NCAM2, NPTXR, NRXN2, RELN, and CNTN2 in impaired cognition patients compared to the intact cognition patients. These five identified biomarker candidates are involved in synaptic function. Putting all together, the present study further substantiate the importance of synaptic dysfunction in the pathophysiology of cognitive decline in aSAH patients.

NCAM2 belongs to the immunoglobulin superfamily of cell adhesion molecules and is essential for proper neuronal differentiation, dendritic and axonal outgrowth and synapse formation (Leshchyns’ka et al., 2015). Although NCAM2 have so far not been associated with cognitive impairment, it has been involved in synaptic deficits in Alzheimer’s disease and was implicated in the intellectual disability phenotype in Down syndrome and autism spectrum disorders, as well as in other neurodevelopmental diseases (Molloy et al., 2005; Petit et al., 2015; Scholz et al., 2016). The present study is the first to report the NCAM2 to be decreased in CSF from aSAH patients with cognitive dysfunction. NPTXR is a member of the neuronal pentraxin family and has been implicated in neuroplasticity, including synaptogenesis (Sia et al., 2007), synaptic plasticity (Xu et al., 2003), metabotropic glutamate receptor-mediated long-term depression, and excitatory and inhibitory synaptic organization (Cho et al., 2008). Chang et al. (2010) reported in 2010 that the reduction of NPTXR is correlated with cognitive decline, implicating a pathophysiological mechanism involving impaired adaptive function of pyramidal neurons. NRXN2 belongs to the neurexin family, a group of synaptic cell adhesion molecules that play key roles in regulating synaptic functions and transsynaptic signaling (Südhof, 2017); it has also been proposed as a CSF biomarker candidate for several neurological disorders. RELN is a gene that encodes a secreted extracellular matrix protein thought to control cell–cell interactions that are critical for the development of the mammalian brain, including dendritic growth, neuronal migration, spine formation, synaptogenesis, and synaptic plasticity. By modulating the formation, function and plasticity of synaptic circuits, RELN directly influences learning and memory (Jossin, 2020). CNTN2 is also a neuronal cell adhesion molecule that encodes a member of the contactin family of proteins and may play a role in regulating neurogenesis as well as the proliferation, migration, and axon guidance of neurons in the developing cerebellum (Ma et al., 2008). Recent studies have demonstrated that CNTN2 may have potential as a biomarker to identify AD patients from healthy controls and may be involved in cognitive flexibility in mice (Huang et al., 2014; Begcevic et al., 2018).

Given the importance of the candidate biomarkers in mediating transsynaptic properties as well as synaptic transmission, our data demonstrating decreased levels of these proteins in the CSF of cognitively impaired aSAH patients at both the mass spectrometry and validation stages, indicating impaired synaptic signaling in these patients. It provides support to the link between cognitive performance and synaptic protein loss in aSAH. On the other hand, learning and memory processes depend on the correct function and number of synapses in the brain (Leshchyns’ka et al., 2015). Based on this, these candidate proteins may be directly involved in the pathophysiological process of cognitive dysfunction in aSAH patients, but more evidence is needed.

Our study has certain limitations. A potential limitation is that the proteomic pipeline is biased toward the identification of more abundant proteins. This is particularly a drawback in mass spectrometry-based proteomic analysis of CSF, since many proteins are secreted from the brain into the CSF, such as cytokines and neuropeptides, at low concentrations. Therefore, we cannot exclude the possibility that we missed some interesting and indispensable proteins. In addition, due to the exploratory nature and the small sample size of the present study, differential proteins between the two groups are presented without adjustments; thus, these findings should be interpreted cautiously and require further confirmation in larger samples. Finally, we did not explore the mechanism by which candidate biomarkers promote cognitive deficits and test its specificity in other diseases samples.

Despite these limitations, this study presents the first deep proteomic investigation of the CSF of aSAH patients with cognitive impairment. We identified and positively validated five novel proteins (NCAM2, NPTXR, NRXN2, RELN, and CNTN2) as promising biomarkers for cognitive impairment following aSAH. Our results suggest that these candidate biomarkers show promise to improve the prediction accuracy for cognitive dysfunction. We anticipate their potential as a treatment target and a future biomarker of disease progression in clinical trials.

## Data Availability Statement

The datasets presented in this study can be found in online repositories. The names of the repository/repositories and accession number(s) can be found below: http://proteomecentral.proteomexchange.org/cgi/GetDataset?ID=PXD032118.

## Ethics Statement

The studies involving human participants were reviewed and approved by Nanfang Hospital of Southern Medical University Institutional Review Board. The patients/participants provided their written informed consent to participate in this study.

## Author Contributions

FL, BQ, and YB performed primary data analysis and wrote the manuscript. JM, XL, and HH are responsible for the collection of CSF. AZ and GZ performed ELISA and statistical analysis of the data. SQ and FM designed and supervised the study. All authors contributed to the article and approved the submitted version.

## Conflict of Interest

The authors declare that the research was conducted in the absence of any commercial or financial relationships that could be construed as a potential conflict of interest.

## Publisher’s Note

All claims expressed in this article are solely those of the authors and do not necessarily represent those of their affiliated organizations, or those of the publisher, the editors and the reviewers. Any product that may be evaluated in this article, or claim that may be made by its manufacturer, is not guaranteed or endorsed by the publisher.

## Footnotes

^1^ http://david.ncifcrf.gov/
